# Supplementary material for: Impacts of Invasive Plants on Native Vegetation Communities in Wetland and Stream Mitigation
Source: Biology (Basel). 2024 Apr 18;13(4):275. doi: 10.3390/biology13040275 (PMC11048567; doi:10.3390/biology13040275)
Supplement: Supplementary file 1 [file biology-13-00275-s001.zip › biology-2937556-supplementary.pdf]

Checklist of Species Sampled (Wetland Dataset)

D. Hunter (sites sampled during peak growing season, 2018)

Scientific Name	Common Name	Family	Status[1]
Acalypha rhomboidea Raf.	Common Three-seeded Mercury	Euphorbiaceae	Native
Acer negundo L.	Eastern Boxelder	Sapindaceae	Native
Acer rubrum L.	Red Maple	Sapindaceae	Native
Acer saccharinum L.	Silver Maple	Sapindaceae	Native
Agalinis purpurea (L.) Pennell	Purple False Foxglove	Orobanchaceae	Native
Agrimonia parviflora Ait.	Small-flowered Agrimony	Rosaceae	Native
Agrostis gigantea Roth	Redtop	Poaceae	Non-native
Alisma subcordatum Raf.	Southern Water-plantain	Alismataceae	Native
Alnus serrulata (Ait.) Willd.	Smooth Alder	Betulaceae	Native
Ambrosia artemisiifolia L.	Common Ragweed	Asteraceae	Native
Ammannia coccinea Rottb.	Scarlet Ammannia	Lythraceae	Native
Ampelopsis brevipedunculata (Maxim.) Trautv.	Porcelain-berry	Vitaceae	Invasive
Amphicarpaea bracteata (L.) Fern.	Hog-peanut	Fabaceae	Native
Anagallis arvensis L.	Scarlet Pimpernel	Primulaceae	Non-native
Andropogon gerardii Vitman	Big Bluestem	Poaceae	Native
Andropogon virginicus L.	Broomsedge	Poaceae	Native
Antennaria plantaginifolia (L.) Richards.	Plantain-leaved Pussytoes	Asteraceae	Native
Apocynum cannabinum L.	Indian Hemp	Apocynaceae	Native
Arthraxon hispidus (Thunb.) Makino	Joint-head Grass	Poaceae	Invasive
Asclepias incarnata L. var. *pulchra* (Ehrh. ex Willd.) Pers.	Swamp Milkweed	Apocynaceae	Native
Azolla caroliniana Willd.	Eastern Mosquito Fern	Salviniaceae	Native
Baccharis halimifolia L.	Groundsel Tree	Asteraceae	Native
Betula nigra L.	River Birch	Betulaceae	Native
Bidens aristosa (Michx.) Britt.	Tickseed Sunflower	Asteraceae	Native
Bidens comosa (Gray) Wiegand	Three-lobe Beggar-ticks	Asteraceae	Native
Boehmeria cylindrica (L.) Sw.	False Nettle	Urticaceae	Native
Campsis radicans (L.) Seem. ex Bureau	Trumpet-creeper	Bignoniaceae	Native
Carex comosa Boott	Bottlebrush Sedge	Cyperaceae	Native
Carex complanata Torr. & Hook.	Hirsute Sedge	Cyperaceae	Native
Carex crinita Lam.	Long-fringed Sedge	Cyperaceae	Native
Carex frankii Kunth	Frank's Sedge	Cyperaceae	Native
Carex grayi Carey	Gray's Sedge	Cyperaceae	Native
Carex lupulina Willd.	Hop Sedge	Cyperaceae	Native
Carex lurida Wahlenb.	Sallow Sedge	Cyperaceae	Native
Carex scoparia Schk. ex Willd.	Broom Sedge	Cyperaceae	Native
Carex squarrosa L.	Squarrose Sedge	Cyperaceae	Native
Carex swanii (Fern.) Mackenzie	Swan's Sedge	Cyperaceae	Native
Carex tribuloides Wahlenb.	Blunt Broom Sedge	Cyperaceae	Native
Carex vulpinoidea Michaux	Fox Sedge	Cyperaceae	Native
Celtis occidentalis L.	Common Hackberry	Cannabaceae	Native
Cephalanthus occidentalis L.	Buttonbush	Rubiaceae	Native
Chasmanthium laxum (L.) Yates	Slender Spikegrass	Poaceae	Native
Cicuta maculata L.	Water-hemlock	Apiaceae	Native

Checklist of Species Sampled (Wetland Dataset)

D. Hunter (sites sampled during peak growing season, 2018)

Scientific Name	Common Name	Family	Status[1]
Cinna arundinacea L.	Common Wood Reedgrass	Poaceae	Native
Coleataenia anceps (Michx.) Soreng	Beaked Panic Grass	Poaceae	Native
Coleataenia stipitata (Nash) LeBlond	Redtop Panic Grass	Poaceae	Native
Conoclinium coelestinum (L.) DC.	Mistflower	Asteraceae	Native
Cornus amomum P. Mill.	Silky Dogwood	Cornaceae	Native
Cuscuta gronovii Willd. ex Roem. & Schult.	Common Dodder	Convolvulaceae	Native
Cyperus bipartitus Torrey	Slender Flatsedge	Cyperaceae	Native
Cyperus difformis L.	Variable Flatsedge	Cyperaceae	Non-native
Cyperus iria L.	Rice-field Flatsedge	Cyperaceae	Non-native
Cyperus pseudovegetus Steudel	Green Flatsedge	Cyperaceae	Native
Cyperus strigosus L.	Straw-colored Flatsedge	Cyperaceae	Native
Desmodium paniculatum (L.) DC.	Narrow-leaf Tick-trefoil	Fabaceae	Native
Dichanthelium clandestinum (L.) Gould	Deer-Tongue Grass	Poaceae	Native
Dichanthelium commutatum (J.A. Schultes) Gould	Variable Panic Grass	Poaceae	Native
Dichanthelium dichotomum (L.) Gould	Small-fruited Panic Grass	Poaceae	Native
Dichanthelium scoparium (Lam.) Gould	Velvet Panic Grass	Poaceae	Native
Digitaria villosa (Walt.) Pers.	Shaggy Crabgrass	Poaceae	Native
Diodia teres Walt.	Common Buttonweed	Rubiaceae	Native
Diodia virginiana L.	Virginia Buttonweed	Rubiaceae	Native
Diospyros virginiana L.	Common Persimmon	Ebenaceae	Native
Echinochloa muricata (Beauv.) Fern. var. *microstachya* Wieg.	Rough Barnyard Grass	Poaceae	Native
Echinochloa muricata (Beauv.) Fern. var. *muricata*	Rough Barnyard Grass	Poaceae	Native
Eclipta prostrata (L.) L.	False Daisy	Asteraceae	Native
Eleocharis acicularis (L.) Roemer & Schultes	Needle Spikerush	Cyperaceae	Native
Eleocharis obtusa (Willd.) Schultes	Blunt Spikerush	Cyperaceae	Native
Eleocharis quadrangulata (Michx.) R. & S.	Square-stem Spikerush	Cyperaceae	Native
Eleocharis tenuis (Willd.) Schultes	Slender Spikerush	Cyperaceae	Native
Elymus virginicus L.	Virginia Wild Rye	Poaceae	Native
Epilobium coloratum Biehler	Purple-leaved Willow-herb	Onagraceae	Native
Erechtites hieraciifolius (L.) Raf. ex DC.	Fireweed	Asteraceae	Native
Erianthus giganteus (Walt.) P. Beauv.	Giant Plumegrass	Poaceae	Native
Eupatorium capillifolium (Lam.) Small	Dog-fennel	Asteraceae	Native
Eupatorium perfoliatum L.	Boneset	Asteraceae	Native
Eupatorium serotinum Michx.	Late Thoroughwort	Asteraceae	Native
Euthamia caroliniana (L.) Greene ex Porter & Britton	Slender Flat-top Goldenrod	Asteraceae	Native
Euthamia graminifolia (L.) Nutt.	Grass-leaved Goldenrod	Asteraceae	Native
Fimbristylis autumnalis (L.) R. & S.	Slender Fimbry	Cyperaceae	Native
Fraxinus pennsylvanica Marsh.	Green Ash	Oleaceae	Native
Galium tinctorium (L.) Scop.	Three-lobed Bedstraw	Rubiaceae	Native
Geum virginianum L.	Cream Avens	Rosaceae	Native

Checklist of Species Sampled (Wetland Dataset)

D. Hunter (sites sampled during peak growing season, 2018)

Scientific Name	Common Name	Family	Status[1]
Hamamelis virginiana L.	Witch Hazel	Hamamelidaceae	Native
Hibiscus moscheutos L.	Swamp Rose-mallow	Malvaceae	Native
Hypericum mutilum L.	Dwarf St. John's-wort	Hypericaceae	Native
Hypericum virginicum L.	Virginia Marsh St. John's-wort	Hypericaceae	Native
Ilex verticillata (L.) Gray	Winterberry	Aquifoliaceae	Native
Impatiens capensis Meerburg	Spotted Jewelweed	Balsaminaceae	Native
Ipomoea lacunosa L.	Small White Morning Glory	Convolvulaceae	Native
Itea virginica L.	Virginia Sweetspire	Iteaceae	Native
Juncus acuminatus Michx.	Sharp-fruited Rush	Juncaceae	Native
Juncus canadensis J. Gay ex Laharpe	Canadian Rush	Juncaceae	Native
Juncus effusus L.	Soft Rush	Juncaceae	Native
Juncus marginatus Rostk.	Grass-leaved Rush	Juncaceae	Native
Juncus tenuis Willd.	Path Rush	Juncaceae	Native
Juniperus virginiana L.	Eastern Redcedar	Cupressaceae	Native
Kummerowia stipulacea (Maxim.) Makino	Korean-clover	Fabaceae	Non-native
Landoltia punctata (G. Mey.) D.H. Les & D.J. Crawford	Dotted Duckmeat	Araceae	Non-native
Leersia oryzoides (L.) Sw.	Rice Cutgrass	Poaceae	Native
Lemna minor L.	Common Duckweed	Araceae	Native
Lespedeza cuneata (Dum.-Cours.) G. Don	Sericea Lespedeza	Fabaceae	Invasive
Lindernia dubia (L.) Pennell var. *anagallidea* (Michx.) Cooperrider	Long-stalked False Pimpernel	Linderniaceae	Native
Lindernia dubia (L.) Pennell var. *dubia*	False Pimpernel	Linderniaceae	Native
Liquidambar styraciflua L.	Sweetgum	Altingiaceae	Native
Liriodendron tulipifera L.	Tulip-tree	Magnoliaceae	Native
Lonicera japonica Thunb.	Japanese Honeysuckle	Caprifoliaceae	Invasive
Ludwigia alata Elliott	Winged Seedbox	Onagraceae	Native
Ludwigia alternifolia L.	Alternate-leaved Seedbox	Onagraceae	Native
Ludwigia decurrens Walter	Wing-leaved Primrose-willow	Onagraceae	Native
Ludwigia glandulosa Walter	Cylindric-fruited Primrose-willow	Onagraceae	Native
Ludwigia palustris (L.) Elliott	Marsh Seedbox	Onagraceae	Native
Lycopus virginicus L.	Virginia Bugleweed	Lamiaceae	Native
Lysimachia nummularia L.	Moneywort	Primulaceae	Invasive
Lythrum salicaria L.	Purple Loosestrife	Lythraceae	Invasive
Microstegium vimineum (Trin.) A. Camus	Japanese Stiltgrass	Poaceae	Invasive
Mikania scandens (L.) Willd.	Climbing Hempweed	Asteraceae	Native
Mimulus alatus Aiton	Winged Monkeyflower	Phrymaceae	Native
Morella cerifera (L.) Small	Wax Myrtle	Myricaceae	Native
Murdannia keisak (Hasskarl) Handel-Mazzetti	Marsh Dewflower	Commelinaceae	Invasive
Nyssa sylvatica Marsh.	Black Gum	Nyssaceae	Native
Onoclea sensibilis L.	Sensitive Fern	Onocleaceae	Native
Oxalis stricta L.	Common Yellow Wood-sorrel	Oxalidaceae	Native
Panicum verrucosum Muhl.	Warty Panic Grass	Poaceae	Native
Panicum virgatum L.	Switchgrass	Poaceae	Native

Checklist of Species Sampled (Wetland Dataset)

D. Hunter (sites sampled during peak growing season, 2018)

Scientific Name	Common Name	Family	Status[1]
Parthenocissus quinquefolia (L.) Planch.	Virginia-creeper	Vitaceae	Native
Paspalum laeve Michx.	Field Paspalum	Poaceae	Native
Peltandra virginica (L.) Schott	Arrow-arum	Araceae	Native
Persicaria arifolia (L.) Haraldson	Halberd-leaf Tearthumb	Polygonaceae	Native
Persicaria glabra (Willd.) M. Gomez	Dense-flowered Smartweed	Polygonaceae	Native
Persicaria hydropiperoides (Michx.) Small	Mild Water-pepper	Polygonaceae	Native
Persicaria pensylvanica (L.) M. Gomez	Pennsylvania Smartweed	Polygonaceae	Native
Persicaria sagittata (L.) H. Gross ex Nakai	Arrow-leaf Tearthumb	Polygonaceae	Native
Phegopteris hexagonoptera (Michx.) Fee	Broad Beech Fern	Thelypteridaceae	Native
Pinus rigida Miller	Pitch Pine	Pinaceae	Native
Pinus taeda L.	Loblolly Pine	Pinaceae	Native
Plantago major L.	Common Plantain	Plantaginaceae	Non-native
Platanus occidentalis L.	Sycamore	Platanaceae	Native
Pluchea odorata (L.) Cass.	Salt Marsh Fleabane	Asteraceae	Native
Poa annua L.	Annual Bluegrass	Poaceae	Non-native
Poa trivialis L.	Rough Bluegrass	Poaceae	Invasive
Pontederia cordata L.	Pickerelweed	Pontederiaceae	Native
Quercus alba L.	White Oak	Fagaceae	Native
Quercus bicolor Willd.	Swamp White Oak	Fagaceae	Native
Quercus michauxii Nutt.	Swamp Chestnut Oak	Fagaceae	Native
Quercus nigra L.	Water Oak	Fagaceae	Native
Quercus palustris Muenchhausen	Pin Oak	Fagaceae	Native
Quercus phellos L.	Willow Oak	Fagaceae	Native
Rhexia mariana L.	Maryland Meadow Beauty	Melastomataceae	Native
Rhynchospora corniculata (Lam.) Gray	Short-bristled Horned Beaksedge	Cyperaceae	Native
Rhynchospora glomerata (L.) Vahl	Clustered Beaksedge	Cyperaceae	Native
Rhynchospora microcephala (Britton) Britton ex Small	Small-headed Bunched Beaksedg	Cyperaceae	Native
Rotala ramosior (L.) Koehne	Toothcup	Lythraceae	Native
Rubus flagellaris Willd.	Common Dewberry	Rosaceae	Native
Rubus pensilvanicus Poir.	Pennsylvania Blackberry	Rosaceae	Native
Rudbeckia laciniata L.	Cut-leaf Coneflower	Asteraceae	Native
Sagittaria latifolia Willd.	Broad-leaved Arrowhead	Alismataceae	Native
Salix nigra Marsh.	Black Willow	Salicaceae	Native
Saururus cernuus L.	Lizard's-tail	Saururaceae	Native
Schoenoplectus mucronatus (L.) Palla	Bog Bulrush	Cyperaceae	Native
Schoenoplectus purshianus (Fern.) Strong	Blunt-scale Bulrush	Cyperaceae	Native
Schoenoplectus tabernaemontani (Gmelin) Palla	Soft-stem Bulrush	Cyperaceae	Native
Scirpus atrovirens Willd.	Dark Green Bulrush	Cyperaceae	Native
Scirpus cyperinus (L.) Kunth	Woolgrass	Cyperaceae	Native
Scirpus georgianus Harper	Georgia Bulrush	Cyperaceae	Native
Scutellaria integrifolia L.	Hyssop Skullcap	Lamiaceae	Native
Scutellaria lateriflora L.	Mad-dog Skullcap	Lamiaceae	Native
Setaria faberi Herrm.	Nodding Bristlegrass	Poaceae	Non-native

Checklist of Species Sampled (Wetland Dataset)

D. Hunter (sites sampled during peak growing season, 2018)

Scientific Name	Common Name	Family	Status[1]
Setaria parviflora (Poir.) Kerguelen	Knotroot Bristlegrass	Poaceae	Native
Setaria pumila (Poir.) Roemer & Schultes	Yellow Bristlegrass	Poaceae	Non-native
Smilax rotundifolia L.	Common Greenbrier	Smilacaceae	Native
Smilax walteri Pursh	Coral Greenbrier	Smilacaceae	Native
Solanum carolinense L.	Horse-nettle	Solanaceae	Native
Solidago altissima L.	Tall Goldenrod	Asteraceae	Native
Solidago rugosa P. Mill.	Wrinkle-leaf Goldenrod	Asteraceae	Native
Sorghum halepense (L.) Pers.	Johnson Grass	Poaceae	Invasive
Symphoricarpos orbiculatus Moench	Coralberry	Caprifoliaceae	Native
Symphyotrichum racemosum (Ell.) Nesom	Small White Aster	Asteraceae	Native
Taxodium distichum (L.) Richard	Baldcypress	Cupressaceae	Native
Toxicodendron radicans (L.) Kuntze	Poison Ivy	Anacardiaceae	Native
Tridens flavus (L.) A.S. Hitchc.	Purpletop	Poaceae	Native
Trifolium pratense L.	Red Clover	Fabaceae	Non-native
Typha latifolia L.	Broadleaf Cattail	Typhaceae	Native
Ulmus rubra Muhl.	Slippery Elm	Ulmaceae	Native
Utricularia geminiscapa Benj.	Two-flowered Bladderwort	Lentibulariaceae	Native
Utricularia gibba L.	Humped Bladderwort	Lentibulariaceae	Native
Verbena hastata L.	Blue Vervain	Verbenaceae	Native
Vernonia glauca (L.) Willd.	Upland Ironweed	Asteraceae	Native
Viburnum dentatum L.	Arrow-wood	Adoxaceae	Native
Viola sororia Willd.	Common Blue Violet	Violaceae	Native
Vitis aestivalis Michx.	Summer Grape	Vitaceae	Native
Vitis rotundifolia Michx.	Muscadine Grape	Vitaceae	Native
Woodwardia areolata (L.) T. Moore	Netted Chain Fern	Blechnaceae	Native
Xanthium strumarium L.	Common Cocklebur	Asteraceae	Native

[1] Status = "Native", "Non-native", or "Invasive" in accordance with Heffernan et al. (2014) and Weakley et al. (2020).

Checklist of Species Sampled (Stream Dataset)

D. DeBerry (sites sampled during peak growing seasons, 2018 and 2019)

Scientific Name	Common Name	Family	Status[1]
Acalypha rhomboidea Raf.	Common Three-seeded Mercury	Euphorbiaceae	Native
Acer rubrum L.	Red Maple	Sapindaceae	Native
Acer saccharinum L.	Silver Maple	Sapindaceae	Native
Aegopodium podagraria L.	Bishop's Goutweed	Apiaceae	Non-native
Agalinis purpurea (L.) Pennell	Purple False Foxglove	Orobanchaceae	Native
Agrimonia parviflora Ait.	Small-flowered Agrimony	Rosaceae	Native
Agrostis perennans (Walt.) Tuckerman	Autumn Bentgrass	Poaceae	Native
Ailanthus altissima (P. Miller) Swingle	Tree-of-heaven	Simaroubaceae	Invasive
Albizia julibrissin Durazz.	Mimosa	Fabaceae	Invasive
Alliaria petiolata (Bieberstein) Cavara & Grande	Garlic Mustard	Brassicaceae	Invasive
Allium canadense L.	Wild Onion	Amaryllidaceae	Native
Alnus serrulata (Ait.) Willd.	Smooth Alder	Betulaceae	Native
Ambrosia artemisiifolia L.	Common Ragweed	Asteraceae	Native
Ambrosia trifida L.	Giant Ragweed	Asteraceae	Native
Amelanchier arborea (Michx. f.) Fernald	Downy Serviceberry	Rosaceae	Native
Ampelopsis brevipedunculata (Maxim.) Trautv.	Porcelain-berry	Vitaceae	Invasive
Amphicarpaea bracteata (L.) Fern.	Hog-peanut	Fabaceae	Native
Andropogon gerardii Vitman	Big Bluestem	Poaceae	Native
Andropogon virginicus L.	Broomsedge	Poaceae	Native
Antennaria plantaginifolia (L.) Richards.	Plantain-leaved Pussytoes	Asteraceae	Native
Artemisia vulgaris L.	Common Mugwort	Asteraceae	Non-native
Arthraxon hispidus (Thunb.) Makino	Joint-head Grass	Poaceae	Invasive
Asclepias incarnata L. var. *pulchra* (Ehrh. ex Willd.) Pers.	Swamp Milkweed	Apocynaceae	Native
Asclepias syriaca L.	Common Milkweed	Apocynaceae	Native
Asimina triloba (L.) Dunal	Pawpaw	Annonaceae	Native
Athyrium asplenioides (Michx.) A.A. Eaton	Southern Lady Fern	Woodsiaceae	Native
Baccharis halimifolia L.	Groundsel Tree	Asteraceae	Native
Betula nigra L.	River Birch	Betulaceae	Native
Bidens aristosa (Michx.) Britt.	Tickseed Sunflower	Asteraceae	Native
Bidens bipinnata L.	Spanish Needles	Asteraceae	Native
Bidens frondosa L.	Devil's Beggar-ticks	Asteraceae	Native
Bignonia capreolata L.	Cross-vine	Bignoniaceae	Native
Boehmeria cylindrica (L.) Sw.	False Nettle	Urticaceae	Native
Botrypus virginianus (L.) Holub	Rattlesnake Fern	Ophioglossaceae	Native
Callicarpa americana L.	American Beauty-berry	Lamiaceae	Native
Calystegia sepium (L.) R. Br.	Hedge Bindweed	Convolvulaceae	Native
Campsis radicans (L.) Seem. ex Bureau	Trumpet-creeper	Bignoniaceae	Native
Cardamine hirsuta L.	Hairy Bittercress	Brassicaceae	Non-native
Carduus nutans L.	Musk Thistle	Asteraceae	Non-native
Carex amphibola Steudel	Eastern Narrow-leaved Sedge	Cyperaceae	Native
Carex blanda Dewey	Eastern Woodland Sedge	Cyperaceae	Native
Carex debilis Michx.	White-edged Sedge	Cyperaceae	Native
Carex frankii Kunth	Frank's Sedge	Cyperaceae	Native

Checklist of Species Sampled (Stream Dataset)

D. DeBerry (sites sampled during peak growing seasons, 2018 and 2019)

Scientific Name	Common Name	Family	Status[1]
Carex lurida Wahlenb.	Sallow Sedge	Cyperaceae	Native
Carex retroflexa Muhl. ex Willd.	Reflexed Sedge	Cyperaceae	Native
Carex squarrosa L.	Squarrose Sedge	Cyperaceae	Native
Carex swanii (Fern.) Mackenzie	Swan's Sedge	Cyperaceae	Native
Carex tribuloides Wahlenb.	Blunt Broom Sedge	Cyperaceae	Native
Carex vulpinoidea Michx.	Fox Sedge	Cyperaceae	Native
Carpinus caroliniana Walt.	Ironwood	Betulaceae	Native
Carya cordiformis (Wangenh.) K. Koch	Bitternut Hickory	Juglandaceae	Native
Carya glabra (P. Miller) Sweet	Pignut Hickory	Juglandaceae	Native
Celastrus orbiculatus Thunb.	Oriental Bittersweet	Celastraceae	Invasive
Celtis occidentalis L.	Common Hackberry	Cannabaceae	Native
Cephalanthus occidentalis L.	Buttonbush	Rubiaceae	Native
Cercis canadensis L.	Eastern Redbud	Fabaceae	Native
Chaerophyllum tainturieri Hook.	Hairy-fruit Chervil	Apiaceae	Native
Chamaecrista fasciculata (Michx.) Greene	Common Partridge-pea	Fabaceae	Native
Chasmanthium latifolium (Michx.) Yates	River Oats	Poaceae	Native
Chasmanthium laxum (L.) Yates	Slender Spikegrass	Poaceae	Native
Chimaphila maculata (L.) Pursh	Spotted Wintergreen	Ericaceae	Native
Cinna arundinacea L.	Common Wood Reedgrass	Poaceae	Native
Circaea canadensis (L.) Hill	Enchanter's Night-shade	Onagraceae	Native
Clematis virginiana L.	Virgin's-bower	Ranunculaceae	Native
Clethra alnifolia L.	Sweet Pepperbush	Clethraceae	Native
Coleataenia anceps (Michx.) Soreng	Beaked Panic Grass	Poaceae	Native
Coleataenia rigidula (Bosc ex Nees) LeBlond	Tall Flat Panic Grass	Poaceae	Native
Commelina communis L.	Asiatic Dayflower	Commelinaceae	Invasive
Conoclinium coelestinum (L.) DC.	Mistflower	Asteraceae	Native
Conyza canadensis (L.) Cronq.	Common Horseweed	Asteraceae	Native
Coreopsis lanceolata L.	Long-stalk Coreopsis	Asteraceae	Native
Cornus amomum P. Mill.	Silky Dogwood	Cornaceae	Native
Cornus florida L.	Flowering Dogwood	Cornaceae	Native
Corylus americana Walt.	American Hazelnut	Betulaceae	Native
Crepis capillaris (L.) Wallr.	Smooth Hawksbeard	Asteraceae	Non-native
Cryptotaenia canadensis (L.) DC.	Honewort	Apiaceae	Native
Cyperus strigosus L.	Straw-colored Flatsedge	Cyperaceae	Native
Dendrolycopodium obscurum (L.) A. Haines	Common Tree-clubmoss	Lycopodiaceae	Native
Desmodium glabellum (Michx.) DC.	Dillenius' Tick-trefoil	Fabaceae	Native
Desmodium paniculatum (L.) DC.	Narrow-leaf Tick-trefoil	Fabaceae	Native
Dichanthelium clandestinum (L.) Gould	Deer-Tongue Grass	Poaceae	Native
Dichanthelium commutatum (J.A. Schultes) Gould	Variable Panic Grass	Poaceae	Native
Dichanthelium dichotomum (L.) Gould	Small-fruited Panic Grass	Poaceae	Native
Dichanthelium laxiflorum (Lam.) Gould	Open-flower Panic Grass	Poaceae	Native
Dichanthelium microcarpon (Muhl. ex Elliott) Mohlenbrock	Branched Panic Grass	Poaceae	Native

Checklist of Species Sampled (Stream Dataset)

D. DeBerry (sites sampled during peak growing seasons, 2018 and 2019)

Scientific Name	Common Name	Family	Status[1]
Dichanthelium scoparium (Lam.) Gould	Velvet Panic Grass	Poaceae	Native
Digitaria ciliaris (Retz.) Koeler	Southern Crabgrass	Poaceae	Native
Digitaria sanguinalis (L.) Scop.	Northern Crabgrass	Poaceae	Non-native
Diodia virginiana L.	Virginia Buttonweed	Rubiaceae	Native
Dioscorea villosa L.	Wild Yam	Dioscoreaceae	Native
Diospyros virginiana L.	Common Persimmon	Ebenaceae	Native
Echinochloa crus-galli (L.) Beauv.	Barnyard Grass	Poaceae	Non-native
Echinochloa muricata (Beauv.) Fern.	Rough Barnyard Grass	Poaceae	Native
Eclipta prostrata (L.) L.	False Daisy	Asteraceae	Native
Elephantopus carolinianus Raeusch.	Carolina Elephant's-foot	Asteraceae	Native
Eleusine indica (L.) Gaertn.	Indian Goosegrass	Poaceae	Non-native
Elymus hystrix L.	Bottlebrush Grass	Poaceae	Native
Elymus virginicus L.	Virginia Wild Rye	Poaceae	Native
Erechtites hieraciifolius (L.) Raf. ex DC.	Fireweed	Asteraceae	Native
Euonymus alatus (Thunb.) Sieb.	Winged Euonymus	Celastraceae	Invasive
Euonymus americanus L.	Strawberry-bush	Celastraceae	Native
Euonymus fortunei (Turcz.) Hand.-Maz.	Winter Creeper	Celastraceae	Invasive
Eupatorium capillifolium (Lam.) Small	Dog-fennel	Asteraceae	Native
Eupatorium perfoliatum L.	Boneset	Asteraceae	Native
Eupatorium rotundifolium L.	Roundleaf Thoroughwort	Asteraceae	Native
Eupatorium serotinum Michx.	Late Thoroughwort	Asteraceae	Native
Euthamia graminifolia (L.) Nutt.	Grass-leaved Goldenrod	Asteraceae	Native
Eutrochium fistulosum (Barratt) E.E. Lamont	Hollow Joe-pye-weed	Asteraceae	Native
Eutrochium purpureum (L.) E.E. Lamont	Sweet-scented Joe-pye-weed	Asteraceae	Native
Fraxinus pennsylvanica Marsh.	Green Ash	Oleaceae	Native
Galium circaezans Michx.	Forest Bedstraw	Rubiaceae	Native
Galium tinctorium (L.) Scop.	Three-lobed Bedstraw	Rubiaceae	Native
Galium triflorum Michx.	Sweet-scented Bedstraw	Rubiaceae	Native
Geum canadense Jacquin	White Avens	Rosaceae	Native
Glechoma hederacea L.	Ground-ivy	Lamiaceae	Invasive
Helenium autumnale L.	Common Sneezeweed	Asteraceae	Native
Hydrocotyle umbellata L.	Marsh Water-pennywort	Araliaceae	Native
Hylodesmum nudiflorum (L.) H. Ohashi & R.R. Mill	Naked-Flowered Tick-trefoil	Fabaceae	Native
Hypericum hypericoides (L.) Crantz	St. Andrew's Cross	Hypericaceae	Native
Hypericum mutilum L.	Dwarf St. John's-wort	Hypericaceae	Native
Hypericum punctatum Lam.	Spotted St. John's-wort	Hypericaceae	Native
Ilex decidua Walt.	Deciduous Holly	Aquifoliaceae	Native
Ilex glabra (L.) Gray	Inkberry	Aquifoliaceae	Native
Ilex opaca Aiton	American Holly	Aquifoliaceae	Native
Ilex verticillata (L.) Gray	Winterberry	Aquifoliaceae	Native
Impatiens capensis Meerburg	Spotted Jewelweed	Balsaminaceae	Native
Ipomoea purpurea (L.) Roth	Common Morning Glory	Convolvulaceae	Non-native
Juglans nigra L.	Black Walnut	Juglandaceae	Native

Checklist of Species Sampled (Stream Dataset)

D. DeBerry (sites sampled during peak growing seasons, 2018 and 2019)

Scientific Name	Common Name	Family	Status[1]
Juncus coriaceus Mackenzie	Leathery Rush	Juncaceae	Native
Juncus dichotomus Ell.	Forked Rush	Juncaceae	Native
Juncus effusus L.	Soft Rush	Juncaceae	Native
Juncus scirpoides Lam.	Needle-pod Rush	Juncaceae	Native
Juncus tenuis Willd.	Path Rush	Juncaceae	Native
Juniperus virginiana L.	Eastern Redcedar	Cupressaceae	Native
Kummerowia striata (Thunb.) Schindl.	Japanese-clover	Fabaceae	Non-native
Laportea canadensis (L.) Weddell	Wood Nettle	Urticaceae	Native
Leersia oryzoides (L.) Sw.	Rice Cutgrass	Poaceae	Native
Leersia virginica Willd.	White Grass	Poaceae	Native
Lespedeza cuneata (Dum.-Cours.) G. Don	Sericea Lespedeza	Fabaceae	Invasive
Ligustrum sinense Louriere	Chinese Privet	Oleaceae	Invasive
Lindera benzoin (L.) Blume	Spicebush	Lauraceae	Native
Liquidambar styraciflua L.	Sweetgum	Altingiaceae	Native
Liriodendron tulipifera L.	Tulip-tree	Magnoliaceae	Native
Lobelia siphilitica L.	Great Blue Lobelia	Campanulaceae	Native
Lonicera japonica Thunb.	Japanese Honeysuckle	Caprifoliaceae	Invasive
Lonicera morrowii Gray	Morrow's Honeysuckle	Caprifoliaceae	Invasive
Ludwigia alternifolia L.	Alternate-leaved Seedbox	Onagraceae	Native
Lycopus americanus Muhl. ex W. Bart.	American Bugleweed	Lamiaceae	Native
Lycopus virginicus L.	Virginia Bugleweed	Lamiaceae	Native
Melia azedarach L.	Chinaberry	Meliaceae	Invasive
Melothria pendula L.	Creeping Cucumber	Cucurbitaceae	Native
Microstegium vimineum (Trin.) A. Camus	Japanese Stiltgrass	Poaceae	Invasive
Mikania scandens (L.) Willd.	Climbing Hempweed	Asteraceae	Native
Mimulus alatus Aiton	Winged Monkeyflower	Phrymaceae	Native
Mimulus ringens L.	Square-stemmed Monkeyflower	Phrymaceae	Native
Miscanthus sinensis Anderss.	Chinese Silvergrass	Poaceae	Invasive
Mitchella repens L.	Partidge-berry	Rubiaceae	Native
Morella cerifera (L.) Small	Wax Myrtle	Myricaceae	Native
Morus rubra L.	Red Mulberry	Moraceae	Native
Muhlenbergia schreberi J.F. Gmel.	Nimblewill	Poaceae	Native
Murdannia keisak (Hasskarl) Handel-Mazzetti	Marsh Dewflower	Commelinaceae	Invasive
Nyssa sylvatica Marsh.	Black Gum	Nyssaceae	Native
Onoclea sensibilis L.	Sensitive Fern	Onocleaceae	Native
Osmorhiza longistylis (Torr.) DC.	Aniseroot	Apiaceae	Native
Osmundastrum cinnamomeum (L.) C. Presl	Cinnamon Fern	Osmundaceae	Native
Oxalis dillenii Jacquin	Southern Yellow Wood-sorrel	Oxalidaceae	Native
Oxalis stricta L.	Common Yellow Wood-sorrel	Oxalidaceae	Native
Packera aurea (L.) A. & D. Love	Golden Ragwort	Asteraceae	Native
Panicum dichotomiflorum Michx.	Fall Panic Grass	Poaceae	Native
Panicum virgatum L.	Switchgrass	Poaceae	Native
Parathelypteris noveboracensis (L.) Ching	New York Fern	Thelypteridaceae	Native
Parthenocissus quinquefolia (L.) Planch.	Virginia-creeper	Vitaceae	Native

Checklist of Species Sampled (Stream Dataset)

D. DeBerry (sites sampled during peak growing seasons, 2018 and 2019)

**Scientific Name**	**Common Name**	**Family**	**Status[1]**
Paspalum floridanum Michx.	Florida Paspalum	Poaceae	Native
Paspalum laeve Michx.	Field Paspalum	Poaceae	Native
Passiflora incarnata L.	Purple Passionflower	Passifloraceae	Native
Penstemon digitalis Nutt. ex Sims	Foxglove Beard-tongue	Plantaginaceae	Native
Penthorum sedoides L.	Ditch Stonecrop	Penthoraceae	Native
Perilla frutescens (L.) Britt.	Beefsteak Plant	Lamiaceae	Invasive
Persicaria hydropiperoides (Michx.) Small	Mild Water-pepper	Polygonaceae	Native
Persicaria lapathifolia (L.) Gray	Dock-leaf Smartweed	Polygonaceae	Native
Persicaria longiseta (Bruijn) Kitagawa	Bristly Lady's-Thumb	Polygonaceae	Invasive
Persicaria pensylvanica (L.) M. Gomez	Pennsylvania Smartweed	Polygonaceae	Native
Persicaria punctata (Ell.) Small	Dotted Smartweed	Polygonaceae	Native
Persicaria sagittata (L.) H. Gross ex Nakai	Arrow-leaf Tearthumb	Polygonaceae	Native
Persicaria virginiana (L.) Gaertn.	Jumpseed	Polygonaceae	Native
Phegopteris hexagonoptera (Michx.) Fee	Broad Beech Fern	Thelypteridaceae	Native
Phryma leptostachya L.	Lopseed	Phrymaceae	Native
Phyllanthus caroliniensis Walt.	Carolina Leaf-flower	Phyllanthaceae	Native
Phytolacca americana L.	Common Pokeweed	Phytolaccaceae	Native
Pilea pumila (L.) Gray	Clearweed	Urticaceae	Native
Pinus taeda L.	Loblolly Pine	Pinaceae	Native
Platanus occidentalis L.	Sycamore	Platanaceae	Native
Pluchea camphorata (L.) DC.	Camphorweed	Asteraceae	Native
Polygonatum biflorum (Walt.) Ell.	Solomon's-seal	Ruscaceae	Native
Polystichum acrostichoides (Michx.) Schott	Christmas Fern	Dryopteridaceae	Native
Populus deltoides Bartram ex Marsh.	Eastern Cottonwood	Salicaceae	Native
Potentilla canadensis L.	Canada Cinquefoil	Rosaceae	Native
Potentilla indica (Andr.) T. Wolf	Indian-strawberry	Rosaceae	Non-native
Prunella vulgaris L.	Heal-all	Lamiaceae	Native
Prunus avium (L.) L.	Sweet Cherry	Rosaceae	Non-native
Prunus serotina Ehrhart	Black Cherry	Rosaceae	Native
Pseudognaphalium obtusifolium (L.) Hilliard & Burtt	Sweet Everlasting	Asteraceae	Native
Pueraria montana (Loureiro) Merritt var. *lobata* (Willd.) Maesen & S. Almeida	Kudzu	Fabaceae	Invasive
Pycnanthemum incanum (L.) Michx.	Hoary Mountain-mint	Lamiaceae	Native
Pycnanthemum tenuifolium Schrad.	Narrow-leaf Mountain-mint	Lamiaceae	Native
Pycnanthemum verticillatum (Michx.) Pers.	Whorled Mountain-mint	Lamiaceae	Native
Pyrus calleryana Dcne.	Callery Pear	Rosaceae	Invasive
Quercus alba L.	White Oak	Fagaceae	Native
Quercus bicolor Willd.	Swamp White Oak	Fagaceae	Native
Quercus falcata Michx.	Southern Red Oak	Fagaceae	Native
Quercus michauxii Nutt.	Swamp Chestnut Oak	Fagaceae	Native
Quercus montana L.	Chestnut Oak	Fagaceae	Native
Quercus palustris Muenchhausen	Pin Oak	Fagaceae	Native
Quercus phellos L.	Willow Oak	Fagaceae	Native

Checklist of Species Sampled (Stream Dataset)

D. DeBerry (sites sampled during peak growing seasons, 2018 and 2019)

Scientific Name	Common Name	Family	Status[1]
Quercus rubra L.	Northern Red Oak	Fagaceae	Native
Ranunculus recurvatus Poir.	Hooked Buttercup	Ranunculaceae	Native
Rhexia mariana L.	Maryland Meadow Beauty	Melastomataceae	Native
Rhexia virginica L.	Virginia Meadow Beauty	Melastomataceae	Native
Rhynchospora glomerata (L.) Vahl	Clustered Beaksedge	Cyperaceae	Native
Robinia pseudoacacia L.	Black Locust	Fabaceae	Native
Rosa multiflora Thunb. ex Murr.	Multiflora Rose	Rosaceae	Invasive
Rosa virginiana Miller	Virginia Rose	Rosaceae	Native
Rubus discolor Weihe & Nees	Himalayan Blackberry	Rosaceae	Non-native
Rubus flagellaris Willd.	Common Dewberry	Rosaceae	Native
Rubus hispidus L.	Bristly Dewberry	Rosaceae	Native
Rubus occidentalis L.	Black Raspberry	Rosaceae	Native
Rubus pensilvanicus Poir.	Pennsylvania Blackberry	Rosaceae	Native
Rudbeckia hirta L.	Black-eyed Susan	Asteraceae	Native
Rumex obtusifolius L.	Bitter Dock	Polygonaceae	Non-native
Salix nigra Marsh.	Black Willow	Salicaceae	Native
Sambucus canadensis L.	Common Elderberry	Adoxaceae	Native
Sanicula canadensis L.	Black Snakeroot	Apiaceae	Native
Saururus cernuus L.	Lizard's-tail	Saururaceae	Native
Sceptridium dissectum (Sprengel) Lyon	Cut-leaf Grape Fern	Ophioglossaceae	Native
Schizachyrium scoparium (Michx.) Nash	Little Bluestem	Poaceae	Native
Scirpus cyperinus (L.) Kunth	Woolgrass	Cyperaceae	Native
Scirpus georgianus Harper	Georgia Bulrush	Cyperaceae	Native
Scirpus polyphyllus Vahl	Leafy Bulrush	Cyperaceae	Native
Scutellaria integrifolia L.	Hyssop Skullcap	Lamiaceae	Native
Senna marilandica (L.) Link	Maryland Wild Senna	Fabaceae	Native
Setaria parviflora (Poir.) Kerguelen	Knotroot Foxtail	Poaceae	Native
Sicyos angulatus L.	Bur Cucumber	Cucurbitaceae	Native
Sida spinosa L.	Prickly Mallow	Malvaceae	Non-native
Silene stellata (L.) Ait. f.	Starry Campion	Caryophyllaceae	Native
Smilax glauca Walt.	Catbrier	Smilacaceae	Native
Smilax rotundifolia L.	Common Greenbrier	Smilacaceae	Native
Solanum carolinense L.	Horse-nettle	Solanaceae	Native
Solidago altissima L.	Tall Goldenrod	Asteraceae	Native
Solidago patula Muhl. ex Willd.	Rough-leaved Goldenrod	Asteraceae	Native
Solidago rugosa P. Mill.	Wrinkle-leaf Goldenrod	Asteraceae	Native
Sorghastrum nutans (L.) Nash	Indian Grass	Poaceae	Native
Sorghum halepense (L.) Pers.	Johnson Grass	Poaceae	Invasive
Stellaria media (L.) Vill.	Common Chickweed	Caryophyllaceae	Invasive
Symphoricarpos orbiculatus Moench	Coralberry	Caprifoliaceae	Native
Symphyotrichum lanceolatum (Willd.) Nesom	Panicled Aster	Asteraceae	Native
Symphyotrichum lateriflorum (L.) A.& D. Love	Calico Aster	Asteraceae	Native
Symphyotrichum novae-angliae (L.) Nesom	New England Aster	Asteraceae	Native
Symphyotrichum novi-belgii (L.) Nesom	New York Aster	Asteraceae	Native

Checklist of Species Sampled (Stream Dataset)

D. DeBerry (sites sampled during peak growing seasons, 2018 and 2019)

Scientific Name	Common Name	Family	Status[1]
Symphyotrichum pilosum (Willd.) Nesom	Frost Aster	Asteraceae	Native
Symphyotrichum puniceum (L.) A.& D. Love	Purple-stem Aster	Asteraceae	Native
Symphyotrichum racemosum (Ell.) Nesom	Small White Aster	Asteraceae	Native
Symphyotrichum undulatum (L.) Nesom	Wavy-leaved Aster	Asteraceae	Native
Teucrium canadense L.	Canada Germander	Lamiaceae	Native
Thelypteris palustris Schott var. *pubescens* (Lawson) Fern.	Marsh Fern	Thelypteridaceae	Native
Toxicodendron radicans (L.) Kuntze	Poison Ivy	Anacardiaceae	Native
Tridens flavus (L.) A.S. Hitchc.	Purpletop	Poaceae	Native
Trifolium repens L.	White Clover	Fabaceae	Non-native
Tripsacum dactyloides (L.) L.	Eastern Gamagrass	Poaceae	Native
Ulmus alata Michx.	Winged Elm	Ulmaceae	Native
Ulmus americana L.	American Elm	Ulmaceae	Native
Ulmus rubra Muhl.	Slippery Elm	Ulmaceae	Native
Uvularia perfoliata L.	Perfoliate Bellwort	Colchicaceae	Native
Vaccinium pallidum Ait.	Early Lowbush Blueberry	Ericaceae	Native
Verbena hastata L.	Blue Vervain	Verbenaceae	Native
Verbena urticifolia L.	White Vervain	Verbenaceae	Native
Verbesina alternifolia (L.) Britt. ex Kearney	Wingstem	Asteraceae	Native
Verbesina occidentalis (L.) Walt.	Yellow Crownbeard	Asteraceae	Native
Vernonia noveboracensis (L.) Michx.	New York Ironweed	Asteraceae	Native
Viburnum dentatum L.	Arrow-wood	Adoxaceae	Native
Viburnum plicatum Thunb.	Japanese Snowball	Adoxaceae	Non-native
Viburnum prunifolium L.	Black Haw	Adoxaceae	Native
Viola sororia Willd.	Common Blue Violet	Violaceae	Native
Vitis labrusca L.	Fox Grape	Vitaceae	Native
Vitis rotundifolia Michx.	Muscadine Grape	Vitaceae	Native
Woodwardia areolata (L.) T. Moore	Netted Chain Fern	Blechnaceae	Native
Xanthium strumarium L.	Common Cocklebur	Asteraceae	Native

[1] Status = "Native", "Non-native", or "Invasive" in accordance with Heffernan et al. (2014) and Weakley et al. (2020).
